# Metabolism at the centre of the host–microbe relationship

**DOI:** 10.1111/cei.13329

**Published:** 2019-06-07

**Authors:** K. M. Maslowski

**Affiliations:** ^1^ Institute of Immunology and Immunotherapy and Institute of Metabolism and Systems Research University of Birmingham Birmingham UK

**Keywords:** autoimmunity, dysbiosis, host‐pathogen interactions, infection, inflammation, physiological hypoxia

## Abstract

Maintaining homoeostatic host–microbe interactions is vital for host immune function. The gut microbiota shapes the host immune system and the immune system reciprocally shapes and modifies the gut microbiota. However, our understanding of how these microbes are tolerated and how individual, or communities of, gut microbes influence host function is limited. This review will focus on metabolites as key mediators of this complex host–microbe relationship. It will look at the central role of epithelial metabolism in shaping the gut microbiota, how microbial metabolites influence the epithelium and the mucosal and peripheral immune system, and how the immune system shapes microbial composition and metabolism. Finally, this review will look at how metabolites are involved in cross‐talk between different members of the microbiota and their role during infections.

## Introduction

The intestinal tract is colonized by a plethora of microbes known collectively as the gut microbiota. From ants to humans, these gut microbes play important roles in maintaining host physiology: from nutrient acquisition and synthesis, energy homoeostasis, maintenance of colonization resistance and immune development and maintenance 1, 2. Dysbiosis, meaning a disruption in the normal microbiota, is associated with a plethora of immune, metabolic and even neurological diseases 3, 4, 5, 6. A common trait in dysbiosis is a shift from obligate anaerobes to facultative anaerobe or aerobic microorganisms 7, 8. Causes of dysbiosis include environmental factors such as antibiotic use and western‐style high‐fat diet 3, but host genetic factors that regulate gut epithelial function and oxygen metabolism, or disease states, may also underlie dysbiosis 3, 7, 8. As such, it is often uncertain whether dysbiosis causes disease or is a result 9. While the understanding of host–microbe interactions has advanced enormously over the past 20 years, there is still much more yet to be defined. With rapidly advancing multiomics technologies we are gaining insight into how metabolism, and metabolites, are key to supporting the host–microbe relationship.

## Colonic epithelial metabolism shapes gut microbial composition and vice versa

The intestinal epithelium is at the interface of host and microbe interactions. Epithelial integrity is vitally important for maintaining host health, as loss of barrier integrity can lead to dissemination of intestinal bacteria, disruption of nutrient acquisition, water loss and activation of mucosal inflammation. Epithelial metabolism has been proposed to be central in establishing the hypoxic environment of the colon lumen that is critical to maintaining obligate anaerobic commensal/mutualistic microbes 7, but the main energy source of colonocytes is butyrate, a metabolic by‐product of the gut microbiota, suggesting that microbiota presence is the first most important step for establishing the host–microbe interface. This cyclical relationship between host epithelial metabolism providing a niche for gut microbes and gut microbes providing an energy source for the epithelium, make it difficult to dissect the underpinning mechanisms at the basis of host–microbe homoeostasis.

Members of the healthy gut microbiota are important for the digestion of complex plant polysaccharides (fibre) from the diet by fermentation and as such, germ‐free mice exhibit an enlarged caecum, due to the build‐up of undigested dietary fibre, and defects in energy harvest – particularly in colonocyte energy homoeostasis 10, 11, 12, 13, 14, 15, 16, 17. Fermentation of dietary fibre results in the production of short chain fatty acid (SCFA), including acetate (C2), propionate (C3) and butyrate (C4). It has long been established that colonocytes preferentially utilize butyrate as an energy source, and doing so utilizes oxygen, with butyrate metabolism accounting for approximately 85% of oxygen consumption by colonocytes 17, 18, 19, 20, 21. Germ‐free mice exhibit decreased epithelial nicotinamide adenine dinucleotide hydrogen (NADH)/NAD^+^ and ATP levels, marked by decreased mRNA and protein expression of various genes involved in metabolism 17, which highlights the importance of the microbiota for epithelial metabolism. Host colonization starts from birth (the fetus is considered largely sterile) and during the first months and years microbes inhabiting the gut undergo successive waves of colonization. First, pioneering aerobic or facultative anaerobes dominate before obligate anaerobes assemble and diversity appears 22, 23, 24. Experiments in germ‐free mice indicate that obligate anaerobes cannot colonize the intestine without prior colonization with aerobic or facultative anaerobes 25, which suggests that these pioneers are involved in establishing a relationship with the host.

**Figure 1:**
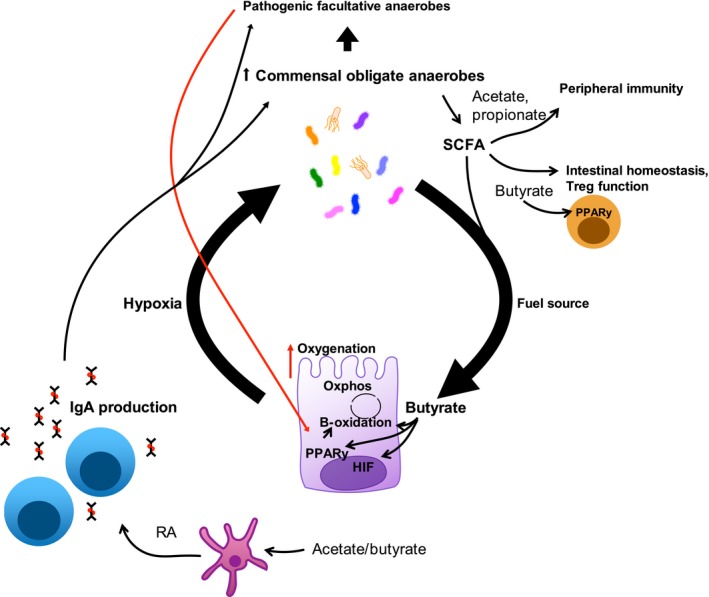
Metabolic interplay between host and gut microbes. Colonic epithelial metabolism of butyrate is central to establishing the host–microbe relationship. Butyrate is a by‐product of fermentation of dietary and host‐derived complex carbohydrates. Certain species of the gut microbiota are responsible for short‐chain fatty acid (SCFA) production (acetate, propionate and butyrate); these are critical members of the community and dysbiosis is often characterized by their loss. Enterocytes at the tip of the villi are main users of butyrate; they express Peroxisome proliferator‐activated receptor gamma (PPARy) (which can be induced by butyrate) which activates β‐oxidation and oxidative phosphorylation. High amounts of oxygen are consumed in this process, making the top of the intestinal crypts physiologically hypoxic. This ensures limited oxygen diffusion into the gut lumen, thereby creating an environment suitable for obligate anaerobes. Hypoxia induces hypoxia‐inducible factor (HIF), which activates a transcriptional network important for maintaining epithelial integrity. SCFA also have a variety of effects on the mucosal and peripheral immune systems, largely acting in a regulatory capacity, limiting inflammatory responses. They also regulate immunoglobulin (Ig)A production via retinoic acid production by dendritic cells, which in turn also modulates microbiota composition.

Several studies have begun to unravel the pathways involved in regulating colonic epithelial metabolism and the interplay between epithelium and gut microbiota. 7, 17, 21, 26, 27, 28. As colonic epithelial cells differentiate they acquire expression of peroxisome proliferator‐activated receptor gamma (PPARγ) 29, which activates fatty acid metabolism enabling mitochondrial β‐oxidation and oxidative phosphorylation with butyrate as the fuel source 17, 27. This utilizes large amounts of oxygen, causing very low levels of oxygen at the mucosal surface (<10 mm/Hg = <1%) 30, 31, 32. Limited oxygen diffusion from the mucosal tissue into the gut lumen makes the hypoxic environment suitable for harbouring obligate anaerobic microbes 7, 27. Obligate anaerobic microbes then preferentially occupy this niche, preventing growth of facultative anaerobes 7. A further layer of complexity is that butyrate itself activates PPARγ 33, making a closed circuit of mutual benefit, but also making it difficult to ascertain where the cycle initially starts (Fig. 1). As described above, obligate anaerobes cannot colonize a germ‐free host without the prior colonization with an aerobic organism. This suggests that early microbial signals may be required to instigate the transcriptional network that activates PPARγ and subsequent high levels of oxygen utilization.

Given that enterocytes at the tip of the colonic crypt exist in a virtually hypoxic environment, they must need to adapt to these conditions to prevent cell death. It has been demonstrated that butyrate metabolism and subsequent oxygen utilization is sufficient to stabilize hypoxia‐inducible factor (HIF) in the colonic epithelium 21. HIF stabilization is important for activating a cellular signature of physiological hypoxia, inducing numerous targets that are involved in maintaining barrier function 34. For example, HIF binds to intestinal trefoil factor (ITF), an important mediator of barrier function 26. Thus, enterocytes are adapted to function and maintain barrier integrity within this physiological hypoxic environment.

Together, these studies suggest that establishment of the transcriptional networks that enable high oxygen consumption by colonocytes and ability to function in physiological hypoxia is critical to establishing the host–microbe relationship, and that early pioneering gut microbes may be required to initiate such transcriptional networks. Perturbations in epithelial metabolism (genetic, disease, damage, other) or perturbations in the gut microbiota (antibiotics, diet, infection, inflammation, other) could disrupt this host–microbe metabolic interdependence. Future studies looking to unravel the complex interplay between host epithelium, microbiota and establishment of this physiological hypoxia may need to look at the events during early host colonization in order to understand more clearly what the first signals are that activate this cyclic interaction between host and microbe. These pathways are less studied in the small intestine, but physiological hypoxia is also apparent in the small intestine 35. Future studies might also look into the effect of fluctuations in blood flow and thereby oxygen abundance, which occurs following feeding. Just a fraction of mucosal capillaries are used under fasting conditions, whereas in the fed state intestinal blood flow can increase up to 200% 35. An interesting question is whether an overfed state, such as that which typically occurs in developed countries where food is abundant, could lead to increased oxygenation of the intestinal villus and lumen leading to altered microbiomes overserved in western cultures, contributing to dysbiosis and disease development or amplification.

## Commensal metabolites and their effects on the intestinal epithelium

The gut microbiota have enormous effects on the host, not only through the presence of microbe‐associated molecular patterns (MAMPs), but also through metabolites they generate. As discussed above, fermentation of dietary fibre results in SCFA production, and butyrate is particularly important for intestinal epithelial homoeostasis. Butyrate also has further effects on epithelial cells through inhibition of histone deacetylases (HDACs) 36 and butyrate, as well as the other SCFAs, acetate and propionate, also have roles in regulating immune functions, which will be discussed later. Aside from SCFA, many other metabolites have been found to directly influence the intestinal epithelium, which will be discussed more here.

Lactic‐acid producing bacteria (LAB) are known to exert a range of beneficial effects on gut homoeostasis and mucosal and systemic immunity 37, 38, 39, 40, but the mechanisms of action are not well characterized. A recent study has shown direct effects of lactate produced by LAB on Paneth cells and submucosal stromal cells via G‐protein coupled receptor 81 (GPR81) 41, a lactate‐specific receptor 42, 43, 44. Production of Wnt ligands by Paneth cells is important for the maintenance of stem cells 45. Intestinal stromal cells and subepithelial mesenchymal cells also express Wnt ligands, and have also been shown to support intestinal stem cells 46, 47. Lee *et al.* 41 found that GPR81 is expressed on Paneth and submucosal stromal cells, and that engagement of lactate induced Wnt ligands resulting in differentiation of intestinal stem cells 41. Functionally, lactate provided by LAB, or supplementation in the drinking water, improved intestinal repair following radiation and chemotherapy‐induced damage when given prophylactically 41. LAB deficient in lactate dehydrogenase, and thereby lactate production, could not exert this beneficial effect, and Gpr81^–/–^ mice have defective intestinal regeneration due to impaired Wnt3 activation 41.

Amino acid metabolism is regulated by the gut microbiota, which affects the amount and type of amino acids available to the host 48, 49, 50. Tryptophan is an essential amino acid important for the generation of a range of bioactive molecules, including serotonin and melatonin 51. The host utilizes the majority of diet‐derived tryptophan, but the gut microbiota also metabolize a portion resulting in indole‐containing molecules 52, 53. Several of these are aryl hydrocarbon receptor ligands and regulate mucosal immunity, which will be discussed in later sections, and is also discussed in detail in a review by Roager and Licht 54.

One commensal, *Clostridium sporogenes*, metabolizes all three aromatic amino acids (AAA), including tryptophan. Analysis of the metabolic pathway involved revealed that there are 12 resulting metabolites, nine of which accumulate in host serum, including indolepropionic acid (IPA) 52. IPA was found to be important for maintaining barrier integrity, and lack of IPA and the other AAA metabolites increased intestinal permeability 52, 55. IPA was shown to bind epithelial pregnane X receptor (PXR), inducing down‐regulation of tumour necrosis factor (TNF)‐α and up‐regulation of junctional protein‐encoding mRNAs 55. Systemic immunity was affected by diminished IPA production, with increased percentage of neutrophils and CD8 T cells and increased anti‐*C. sporogenes* immunoglobulin (Ig)A 52. This work demonstrates that active maintenance of the intestinal barrier, as instructed by microbial metabolites, is critical for maintaining the host tolerance of the gut microbiota.

Tryptamine is a tryptophan‐derived monoamine, which has structural similarity to 5‐hydroxytryptamine (serotonin) and is produced by gut microbes. Bhattarai *et al.* 56 observed that microbial‐derived tryptamine activates the 5‐HT_4_ receptor (5‐HT_4_R) on the intestinal epithelium, increasing colonic secretion and decreasing the time taken for gastric emptying (transit time). Thus, this microbial derivative of tryptophan may have important roles in regulating responses to infection which rely on intestinal secretions to flush out the pathogen 56. Tryptamine, or bacteria engineered to produce tryptamine, may also represent a novel treatment for diseases distinguished by slow transit time, such as irritable bowel syndrome (IBS). Pharmacological agonists of the 5‐HT_4_R have been trialled for IBS; however, significant side effects can occur due to activation of this receptor on other cell types, such as heart muscle cells. Thus, specific delivery of a 5‐HT_4_R ligand to the gut would be a desirable treatment option 56.

## Effect of commensal metabolites on host immune function

Microbial diversity has been identified as an important factor in mediating immune development and maintenance of homoeostasis. Many inflammatory and autoimmune diseases are associated with dysbiosis, but whether dysbiosis is the cause or consequence of these diseases is difficult to dissect. In any case, dysbiosis at least acts to amplify inflammation 9. Sequencing of human microbiomes has revealed a wide variation in species composition, thus making it difficult to ascribe beneficial, or detrimental, effects to specific species. Instead, it is emerging that common functional activities and metabolites are conserved between individuals. Thus, it appears that functional metabolic niche fulfilment is more important than specific individual species within the microbiota 9, 57, 58. Numerous metabolites present in the circulation are dependent on the presence of gut microbiota 53; thus, it is not surprising that the microbiota can exert effects on the host beyond the gut, including effects on host metabolism, the endocrine system, brain and peripheral immune and inflammatory responses 3, 5, 6, 58, 59, 60. This review will focus on a few examples of microbial metabolites affecting host mucosal and peripheral immune responses.

Dysbiosis is commonly associated with a shift towards aerobes and facultative anaerobes and decreased levels of SCFAs 4, 7, 61, 62. SCFAs, acetate, propionate and butyrate, were found to bind G‐protein coupled receptors GPR41 and 43 with varying affinity 63, 64. In a landmark paper, we described a diet–microbiota–host immune axis where microbial metabolism of dietary fibre resulted in SCFA production, and acetate in particular activated GPR43 on innate immune cells, including eosinophils and neutrophils 65. Acetate ligation of GPR43 on neutrophils affected chemotaxis, reactive oxygen species (ROS) and phagocytic activity. In the absence of microbiota, colitis and arthritis was exacerbated, and this could be rescued by addition of acetate in the drinking water 65. Mice lacking Gpr43 also exhibited exacerbated colitis, arthritis and allergic airway responses, which could not be rescued by addition of acetate to the drinking water 65. Numerous other effects of SCFA, whether receptor‐dependent or ‐independent have now been reported 66, 67, 68, 69.

Antibiotic treatment‐induced dysbiosis can have wide‐reaching effects, for example on inflammation, infection and responses to vaccination 70, 71, 72. Antibiotics have been shown to affect SCFA‐producing bacteria causing a decrease in SCFA abundance 73, 74. In a recent study, macrophage dysfunction was noted following antibiotic‐induced dysbiosis causing aberrant activation of T helper type 1 (Th1) cells, leaving mice susceptible to Th17 and Th2‐type infections 75. The authors demonstrated that SCFA were reduced following antibiotics, and that supplementing butyrate could restore macrophage hyporesponsiveness to microbial ligands and prevent Th1 over‐activation 75, thus showing a wider effect of antibiotics on the host beyond microbial composition and effects on colonization resistance. This observation is likely to also be applicable to conditions where SCFA are known to have peripheral effects and also suggest that antibiotic treatment would affect other microbial‐derived metabolites.

Acetate and butyrate have also been found to affect IgA responses. In two separate studies, acetate was found to affect CD103^+^ dendritic cell retinol dehydrogenase activity resulting in increased retinoic acid (RA) production, which increased intestinal IgA production 66, 76. Tan *et al.* additionally showed enhanced T follicular helper cell function supporting mucosal germinal centre reactions 66. This enhanced mucosal IgA production was important for controlling food allergy responses, with zero fibre feeding, or knock‐outs of Gpr43 or Gpr109A (butyrate receptor), being more susceptible to food allergy 66.

Within the mucosal tissue, SCFAs, butyrate in particular, is important for maintaining immune homoeostasis through induction of regulatory T cells (T_regs_) 77, 78. Butyrate‐stimulated T_reg_ expansion in the lamina propria and bone marrow has been shown to promote interaction between bone marrow T_regs_ and CD8 T cells, increasing production of Wnt10b by CD8 T cells, which has effects on stromal cells and osteoclasts promoting bone formation 79. Supplementation of *Lactobacillus rhamnosus* GG (LGG) had previously been shown to affect bone formation, but the mechanisms had not been described. In this study, they confirm that LGG increases bone mass via increased circulation of butyrate. Interestingly, LGG itself is not responsible for butyrate production, but promotes other bacterial species that produce butyrate 79, again highlighting the complex interplay between different microbial communities and how a particular species may not be the key dominant factor, but rather that fulfilment of a metabolic niche is important for maintaining homoeostasis.

The maternal gut microbial composition and products, particularly SCFA, have been shown to affect immune development in offspring 80, 81. In one study, high‐fibre feeding or supplementation with acetate reduced susceptibility to allergic airway disease (AAD) in mice, and furthermore could supress AAD development in the offspring of mice fed on a high‐fibre/acetate diet during pregnancy 80. This, they showed, had an effect on T_reg _expression of forkhead box protein 3 (FoxP3) and blocking T_regs_ ablated the beneficial effects of acetate on AAD development 80. Nakajima *et al.* 82 have shown that microbial‐derived butyrate affected thymic expression of Aire, which is known to be important for T_reg_ selection in the thymus. In further studies, they demonstrated that maternal delivery of butyrate increased thymic expression of Gpr41 and Aire and increased thymic and peripheral T_reg_ numbers 81. This study did not determine if the altered T_reg_ number had any effect on peripheral immune responses but, coupled with the other studies described above, the data would suggest that SCFA can affect T_reg_ development via a multitude of mechanisms 77, 78, 79, 80, 81.

While many studies have found effects of SCFA, other microbial‐derived metabolites are also being discovered to affect immune responses. Morita *et al*. 83 found that GPR31 expression on CX3CR1^+^ intestinal dendritic cells senses bacterial‐produced lactate and pyruvate and mediates dendrite extension and luminal sampling. Supplementation of lactate or pyruvate enhanced uptake and immune responses to *S*. *typhimurium* infection, which was not afforded in Gpr31^–/–^ mice. Engagement of lactate with its other receptor, Gpr81, on intestinal dendritic cells and macrophages was also shown to reduce colitis by suppressing cytokine production and Th1/Th17 cell differentiation 84, but whether this was due to gut microbe‐generated lactate in particular was not addressed.

## Effect of the host immune system on composition of the gut microbiota

IgA plays a critical role in controlling the composition and diversity of the intestinal microbiota 85, 86. The importance of maintaining bacterial composition by IgA is highlighted by the fact that people with selective IgA deficiency have a higher predisposition to autoimmune diseases 9, 87. IgA not only limits enteric pathogens 88, 89, but also actively promotes beneficial microbes. For example, IgA coating enhances *Bacteroides fragilis* habitation of the mucous and mediates intimate interactions with the intestinal epithelium 90.

A recent study showed further importance of IgA coating of a strain of *Bacteroidetes*, which was important for modulating interphylum bacterial interactions and co‐operation via shared metabolism 91. Aiming to test whether glycan–glycan interactions between IgA, bacteria and mucous are important for modulating gut microbiota, Nakajima *et al.* 91 developed a highly glycosylated anti‐ovalbumin IgA monoclonal antibody. This antibody bound preferentially to metabolically active members of the *Bacteriodales* via glycan‐lipopolysaccharide (LPS) interactions. Binding of this highly glycosylated, antigen‐independent antibody altered microbial expression of polysaccharide utilization loci (PUL) genes, particularly in mucosal‐associated bacteria, and not bacteria present in the colonic content 91. Highly expressed PUL genes included components of the starch utilization system, which the authors propose act as symbiotic factors enabling bacterial presence in the mucous environment, and have provisionally named them as mucous‐associated functional factors (MAFFs) 91. A complex interaction between *B. thetaiotaomicron* in the mucous, expression of MAFFs and presence of other symbiotic partners was required for the full effect of MAFFs. IgA binding‐induced expression of MAFFs drove the expansion of butyrate‐producing *Clostridiales* and promoted colonic homoeostasis protecting in a model of colitis 91. IgA has been known for some time to be important in regulating gut microbial composition 63, but this study is the first to describe such complex interplay between a host factor (IgA), microbial selection in a given niche and interphylum microbial interaction, resulting in metabolites that benefit the host.

IgA has also been suggested to limit microbial metabolite penetration, thus impacting on the host response to microbial metabolites 92. Uchimura *et al.* 92 colonized germ‐free mice with a replication‐deficient *Escherichia coli* which had been grown in ^12^C‐labelled medium, enabling them to trace metabolites that originated from the bacteria within tissues and urine. Because of the transient colonization, they could compare between mice that had received no prior colonization and those that had and therefore had IgA induction. They found that IgA promoted the clearance of metabolites from the host tissues by accelerating microbial clearance 92. While this study was able to show that IgA affects the distribution of metabolites in this setting, which would be in agreement with other studies showing the importance of IgA in selecting bacterial species and affecting the time bacteria reside in the gut 85, 86, 89, whether the same kind of effects would be applicable in the more complex setting of a colonized gut is uncertain. For example, several studies show that IgA can increase the dwell time of certain bacteria, which can have follow‐on effects on the wider microbial composition and metabolite availability 90, 91. Nevertheless, all these studies demonstrate the importance of IgA in the regulation of the microbiota composition, which thereby affects metabolic activity of the microbiome and metabolites released into the host system.

## Metabolic interplay between host, microbiota and pathogens

Some of the studies discussed above describe examples of interphylum co‐operation via metabolites. This section expands on that theme and will highlight some examples of co‐infections relying on metabolic subversion, or utilization of certain metabolites, to aid infection or promote host responses.

A type 2 innate immune circuit between the microbiota, intestinal tuft cells and group 2 innate lymphoid cells (ILC2s) has been described by several groups 93, 94, 95. Intestinal tuft cells are a population of chemosensory cells that can be found in the airways, trachea and intestinal tract 96. In the small intestine they have been described to produce interleukin (IL)‐25 upon helminth infection. This initiates IL‐13 production by ILC2s, which feeds back on epithelial progenitor cells and biases their differentiation into goblet and tuft cells, thus promoting a positive feed‐forward loop that promotes worm clearance 93, 94, 95. Recent reports found that the tuft cell–ILC2 circuit could be induced by microbial‐derived succinate binding to G‐protein coupled receptor 91 (GPR91, also known as succinate receptor 1, SUCNR1) 97, 98, 99, 100. SUCNR1 is specifically expressed on small intestinal tuft cells, and ligation with succinate induces a downstream chemosensing pathway involving α‐gustducin (Gnat3) and Trpm5. Supplementing succinate in the drinking water was sufficient to activate SUCNR1 and induce the IL‐25–ILC2–IL‐13‐driven expansion of goblet and tuft cells. Succinate produced by the protist *Tritrichomonas* also drove a type‐2 response through SUCNR1 signalling. In the context of helminth infection, however, SUCNR1 signalling appeared redundant or absent, although downstream activation of Trpm5 and IL‐25 occurs 99, 100, although one of these studies showed that activation of this type 2 circuit by *Tritrichomonas*‐derived succinate protected from subsequent infection with helminths 97. This would suggest that the expansion of goblet and tuft cells is important for limiting infection with helminths. Even though helminths may be able to produce succinate during infection, helminth‐derived succinate does not induce SUCNR1 signalling. This may be due to suppression of succinate production, use of alternative pathways or perhaps localization of succinate production.

Several studies have shown the important effects of aryl hydrocarbon receptor (AHR) ligands on the gut epithelium, maintaining the stem cell niche, barrier function and protection from tumorigenesis and infection 101, as well as important effects on immune cells and peripheral inflammatory responses 102, 103. AHR ligands also regulate mucosal immunity through induction of IL‐22, a cytokine important for epithelial restitution, induction of anti‐microbial peptides and regulation of inflammation 104. Zelante *et al.* 105 found that tryptophan metabolism by stomach‐resident commensal *Lactobacillus reuteri* resulted in indole‐3‐aldehyde production (IAld), which they showed activated AHR signalling in NKp46^+^ cells (and possibly also other cell types) which increased IL‐22 production by stomach epithelia. This, they demonstrated, was important for resistance against *Candida albicans* infection 105. Increasing tryptophan supply by dietary supplementation, or deletion of host indolamine 2,3‐dioxygenase 1 (IDO1), increased levels of the tryptophan metabolite IAld specifically (due to enzymatic pathways present in *L. reuteri*), which mediated these effects 105.

Caspase recruitment domain‐containing protein 9 (CARD9) is a signalling adaptor protein involved in integrating signals from a range of innate receptors, including C‐type lectin and nucleotide‐binding oligomerization domain‐like (NOD) receptors, and is thus involved in bacterial, viral and fungal immune responses 106. CARD9 deficiency was found to affect gut microbial composition and had a resultant effect on tryptophan metabolism 107. Reductions in certain bacterial populations, including *L. reuteri* and *Allobaculum* sp., was associated with diminished indole‐3‐acetic acid (IAA) production in the colon 107. Accordingly, faeces from CARD9^–/–^ mice had reduced ability to activate an AHR reporter, indicating the potential of microbial‐derived IAA to signal through AHR. CARD9^–/–^ mice were more susceptible to colitis, in concordance with CARD9 being a risk allele for IBD in humans. Adding an AHR agonist rescued IL‐22 and adenosine monophosphate (AMP) production and protected from colitis in CARD9^–/–^, again showing the importance of AHR ligands in promoting IL‐22 production and epithelial barrier function. Furthermore, IL‐22 itself alters microbial composition, particularly reducing *Lactobacillus* sp. 107, 108, probably through the production of AMPs, and thereby affects tryptophan metabolism, forming a cyclical relationship between microbial tryptophan metabolism, AHR agonism, IL‐22 production and modulation of the intestinal epithelium 107.

IL‐22 production is increased upon enteric infection and is protective against some pathogens, such as *Citrobacter rodentium*, and colitis, as discussed above and elsewhere 107, 109, 110, 111. However, IL‐22 can promote colonization by other pathogens such as *S*. *typhimurium* by controlling growth of its niche competitor *E. coli* 112. In a study by Grizotte‐Lake *et al.* 113 they found that commensal microbes suppress small intestinal epithelial expression of Rdh7, an enzyme required for retinoic acid (RA) production, resulting in reduced RA in SPF mice compared to germ‐free mice. This effect was due to spore‐forming *Clostridia* sp. The reduction in RA in SPF mice resulted in diminished IL‐22 production by ILC3s and other T cell populations. To understand the effects of Rdh7, they used Rdh7^–/–^ mice and could recapitulate the reduction in RA and IL‐22. This resulted in decreased production of AMPs by the small intestinal epithelium which led to alterations in the gut microbiota, and promoted host resistance to *S. typhimurium* infection (there was no effect on *C. rodentium* infection). Along with the studies described above 107, 108, this suggests that a degree of tonic regulation of IL‐22 mediated by the gut microbiota is important for modulating gut microbial composition and also susceptibility to intestinal pathogens. This highlights how these networks need to be finely tuned in order to promote homoeostatic interactions between host and commensals while limiting pathogens.

Pathogens can alter the gastrointestinal environment to enable them to thrive. *Salmonella* spp. virulence factors induce inflammation in the mucosa characterized by neutrophil recruitment. Neutrophils, and the inflammation induced, release a large amount of electron acceptors, such as tetrathionate and nitrate, which fuel *Salmonella* metabolism, allowing their expansion while altering the gut microbial composition, including the depletion of *Clostridia* spp. 114, 115. This causes a reduction in butyrate which alters metabolic activity of enterocytes which usually rely on butyrate, as discussed above, and thus leads to increased oxygen availability, further supporting *Salmonella* growth 7. Changes in enterocyte metabolism were found to generate lactate which, along with the increase in oxygen, *Salmonella* could use as an energy source with oxygen as the final electron acceptor 70. Furthermore, it was recently reported that *Salmonella* can utilize microbiota‐derived succinate and host‐derived electron acceptors to enable a complete oxidative tricarboxylic acid (TCA) cycle 116. Thus, pathogens such as *Salmonella* spp. can actively induce dysbiosis, or dysbiosis through other causes can support the growth of *Salmonella*.

More complex interactions between host and microbial species can occur in co‐infection settings. Chronic helminth infections are associated with increased occurrence of co‐infections with a range of bacterial and viral pathogens 117, 118, 119, 120. Induction of type 2 (Th2) immune responses and T_regs_ is thought to play a major role in limiting protective Th1 and Th17 responses required to clear bacterial and viral pathogens 118, 119, 120, 121. Helminth infections can alter the microbial composition and have been shown to increase SCFA production, which can alleviate allergic airway inflammation and colitis 122, 123. Using a co‐infection model, Reynolds *et al.* 124 found that helminth infection (*Heligmosomoides polygyrus*) altered the metabolome of small intestinal epithelial cells, which has a resultant effect on *Salmonella* expression of invasion genes within the *Salmonella* pathogenicity island‐1 (SPI‐1). Metabolites extracted from naive small intestinal epithelial cells could supress SPI‐1 expression and reduce invasion of *Salmonella* in an *in‐vitro* invasion assay, whereas helminth‐infected epithelial metabolites could not, and thus increased *Salmonella* invasion was observed if mice were already infected with helminths 124. This study did not identify individual metabolites that exert this effect, so whether individual or multiple metabolites are responsible is yet to be determined. Given that helminths, or helminth‐derived products, are of interest as therapeutic interventions for allergic airway and inflammatory bowel disease, it is important to understand possible negative impacts they may have in promoting co‐infections 124.

Altogether, these studies highlight the complex interactions between host and microbes, as well as intermicrobial interactions, many of which are mediated by, or involve subversion of, metabolic pathways and metabolites. Epithelial metabolism and physiological hypoxia are central to the host–microbe relationship. As colonocytes are differentiated at the top of the crypts they express PPARγ, which switches on β‐oxidation of butyrate and oxidative phosphorylation which consumes oxygen. By the induction of physiological hypoxia, HIF becomes stabilized and then induces transcription of essential genes required for epithelial barrier function, such as tight junction proteins, anti‐microbial peptides and mucous production. The hypoxic environment created then favours obligate anaerobes which act in a mutualistic relationship, providing the main energy source, butyrate, for the colonocytes by fermentation of dietary fibre (Fig. 1). This establishes obligate anaerobes as the dominant bacteria within the colon, and further metabolites derived from the microbiota, such as acetate, lactate, AHR ligands, indole‐derived molecules, etc. are now appreciated to exert a broad range of effects on immune and host function, as well as feeding back to select the microbiome further via IgA or anti‐microbial peptides. These processes, from epithelial metabolism to maintenance of obligate anaerobes, are disrupted during infections, even purposefully, favouring facultative anaerobes or aerobes, which can have deleterious effects on the host.

Future considerations in this area would be to understand more clearly the development of epithelial metabolic pathways, understand any differences between small intestine and the colon and how fluctuations in oxygen could be affecting the microbiome and therefore host function. As discussed above, constant feeding leading to increased blood flow and oxygenation in the intestinal tract could be deleterious and one way by which a western lifestyle may be causing dysbiosis and increased susceptibility to inflammatory diseases. It is also of interest that chronic bowel inflammation, such as in Crohn's disease or ulcerative colitis, causes increased angiogenesis in the intestinal tract, and thus increased oxygen availability might be one reason for poor treatment outcomes in many patients, where dysbiosis and ongoing inflammation are concomitant factors 125. In addition to increased angiogenesis, intestinal inflammation is also associated with bleeding in to the gut lumen, which also delivers increased haem‐bound oxygen. As such, targeting oxygen has been proposed as a treatment option for inflammatory bowel diseases 8.

Some obligate anaerobic commensals cope with oxygen by employing mechanisms such as extracellular electron shuttles, which add electrons to oxygen. This relies upon flavins and thiols being present in the gut; however, during inflammation thiols become depleted 8, 126. Faecal microbiota transplant has been employed successfully for *C. difficile*‐infected patients, and is entering use for further conditions from inflammatory bowel disease to asthma and cancer treatment. However, defining ‘good’ faecal donors is a challenge, and presence of microbial species that are good colonizers and have traits such as being able to utilize and deplete oxygen may be an important consideration.

In summary, metabolism and metabolites are the basis of host–microbe interactions. Further understanding of these metabolic interactions will be important for developing treatment strategies for inflammatory bowel diseases as well as the wide range of other diseases associated with dysbiosis.

## Disclosures

The author declares no conflict of interest.
